# A recombinant chimera comprising the R1 and R2 repeat regions of *M. hyopneumoniae* P97 and the *N*-terminal region of *A. pleuropneumoniae* ApxIII elicits immune responses

**DOI:** 10.1186/1746-6148-10-43

**Published:** 2014-02-18

**Authors:** Seung Heon Lee, Seungwoo Lee, Chanhee Chae, Doug-Young Ryu

**Affiliations:** 1 College of Veterinary Medicine, Seoul National University, 1 Gwanak-ro, Gwanak-gu, Seoul 151-742, South Korea

**Keywords:** *Actinobacillus pleuropneumoniae*, ApxIII, *Mycoplasma hyopneumoniae*, P97, Recombinant chimera, Vaccine

## Abstract

**Background:**

Infection by *Mycoplasma hyopneumoniae* and *Actinobacillus pleuropneumoniae*, either alone or together, causes serious respiratory diseases in pigs.

**Results:**

To develop an efficient multi-disease subunit vaccine against these pathogens, we produced a chimeric protein called Ap97, which comprises a deletion derivative of the *N*-terminal region of the *A. pleuropneumoniae* ApxIII toxin (ApxN) and the R1 and R2 repeats of *M. hyopneumoniae* P97 adhesin (P97C), using an *E. coli* expression system.

The levels of both IgG1 and IgG2a isotypes specific for ApxN and P97C in the sera of Ap97-immunized mice increased, and Ap97 induced the secretion of IL-4 and IFN-γ by mouse splenocytes. Antisera from mice and pigs immunized with Ap97 readily reacted with both native ApxIII and P97 proteins. In addition, immunization with the Ap97 vaccine effectively protected pigs against challenge with both pathogens.

**Conclusions:**

These findings suggest that Ap97 confers immunogenicity, and is an effective vaccine that protects pigs against infection by *M. hyopneumoniae* and *A. pleuropneumoniae*.

## Background

*Actinobacillus pleuropneumoniae* is the etiological agent responsible for porcine pleuropneumonia, a contagious and potentially fatal respiratory disease in pigs [1-3]. Based on differences in the capsular polysaccharides, 15 serotypes of *A. pleuropneumoniae* have been identified to date, which show significant variation with respect to virulence and geographical distribution. Most of those serotypes secrete one or more *A. pleuropneumoniae* toxin (Apx), identified as ApxI, ApxII, ApxIII, and ApxIV. Apx toxins are major virulence factors of *A. pleuropneumoniae* and are highly immunogenic. It is well accepted that ApxI and ApxIII are highly toxic for porcine neutrophils and pulmonary alveolar macrophages, and ApxII only moderately so.

*A. pleuropneumoniae* vaccines include inactivated whole-cell bacterins as well as the more promising subunit vaccines and live attenuated vaccines [4]. Most commercially available vaccines against *A. pleuropneumoniae* infection comprise inactivated whole-cell bacterins derived from various serotypes of *A. pleuropneumoniae*[5]. However, bacterins confer only partial protection against homologous serotypes and do not usually protect against challenge with heterologous serotypes [6,7]. In addition, a large number of mutants were generated and used to produce live attenuated vaccines [8-10]. However, live attenuated vaccines present a number of bio-safety concerns.

Many studies show that subunit vaccines elicit an effective immune response and provide protection against *A. pleuropneumoniae* infection. Apx proteins have been studied as potential candidates for the development of vaccines against porcine pleuropneumoniae because they are highly immunogenic. Many commercially available *A. pleuropneumoniae* subunit vaccines contain recombinant Apx proteins [11]. In addition, chimeric proteins containing components of Apx toxin have been produced for vaccine development. Some epitopes of ApxIA were inserted into the B subunit of *E. coli* heat-labile enterotoxin, and the immunogenicity of the chimeric proteins was analyzed in mice [12]. Recently, ApxIIA fused with the cholera toxin B subunit was expressed in corn as a subunit vaccine candidate [13].

*Mycoplasma hyopneumoniae* is the etiological agent responsible for porcine enzootic pneumonia, a chronic non-fatal disease that affects pigs of all ages [14]. Pigs with primary mycoplasmal infection are predisposed to potentially fatal secondary infections by the porcine reproductive and respiratory syndrome virus, *Pasteurella multocida*, and *A. pleuropneumoniae*[15-17].

Commonly used vaccines against *M. hyopneumoniae* comprise inactivated whole-cell bacterins. Although bacterin-based vaccines often reduce the infection level in a herd, protection against clinical pneumonia is usually not complete [18,19]. In an experimental transmission study, a bacterin vaccine reduced the transmission of *M. hyopneumoniae* only to a limited extent [20]. Moreover, preparation of bacterin vaccines is not economically advantageous, because *in vitro* culture of *M. hyopneumoniae* is time-consuming and requires a rich medium [21].

P97 adhesin is highly conserved among different strains of *M. hyopneumoniae*[22,23]. P97 is essential for the adherence of *M. hyopneumoniae* to ciliated respiratory epithelial cells. The *C*-terminus of P97 contains two repeat regions, designated R1 and R2, which play key roles in adherence [24,25]. Following the discovery of a functional role for the *C*-terminus of P97, much effort has been made to develop vaccines based on this region, in particular the R1 and R2 repeats.

A recombinant chimera comprising the R1 region of P97 and *E. coli* enterotoxin B induced increased humoral and cellular immune responses in mice when compared with the recombinant R1 region alone [26]. Another chimera comprising the R1 region and a domain of *Pseudomonas* exotoxin A stimulated a higher IgG response to the R1 region than a commercial *M. hyopneumoniae* vaccine [27].

Live bacteria harboring vectors encoding either R1, or R1 and R2, have also been evaluated for their potential as vaccines against *M. hyopneumoniae* infection. Mice that orally ingested *Salmonella typhimurium* harboring eukaryotic or prokaryotic expression vector encoding the R1 region showed R1-specific Th1 responses [28]. Intranasal and oral immunization of *Erysipelothrix rhusiopathiae* strains expressing the *C*-terminal portion of P97, including the R1 and R2 regions, to pigs reduced the severity of pneumonic lung lesions caused by *M. hyopneumoniae* infection [29,30]. Adenovirus expressing the *C*-terminal portion of P97, containing the R1 and R2 regions, also induced protective P97-specific humoral and cellular immune responses in pigs [31].

There is a high demand for multi-disease vaccines in the swine industry due to the high labor costs incurred by vaccination and because of injection-induced stress in pigs. This is particularly true for preventing *M. hyopneumoniae* infection and/or secondary infection induced by *A. pleuropneumoniae*[16]. Therefore, the aim of the present study was to produce a chimeric fusion protein comprising the *N*-terminal portion of ApxIII and the *C*-terminal portion of P97 using an *E. coli* expression system, and to analyze its immunogenicity and protective effects against infection by *M. hyopneumoniae* and *A. pleuropneumoniae*.

## Methods

### Chemicals

All chemicals were of reagent grade or higher and were obtained from Sigma-Aldrich (St. Louis, MO), unless otherwise specified.

### Microbial culture

*A. pleuropneumoniae* strain 1536 (serotype 2) and *M. hyopneumoniae* strain J were purchased from the American Type Culture Collection (ATCC, Manassas, VA). A wild-type strain of *M. hyopneumoniae* was isolated from the lung tissues of pigs suffering from porcine enzootic pneumonia.

The *A. pleuropneumoniae* strain was cultured in pleuropneumonia-like organism (PPLO) media (BD Biosciences, Sparks, MD) supplemented with 10 μg/ml nicotinamide dinucleotide, 260 μg/ml L-cysteine hydrochloride, 1 mg/ml dextrose, 10 μg/ml L-cysteine dihydrochloride, 0.6 mM glutamine, and 0.1% Tween 80 at 37°C with continuous shaking [32]. For whole-cell lysate preparations, *A. pleuropneumoniae* was cultured to mid-log phase and collected by centrifugation at 3,000 × g for 10 min. The cell pellet was washed with phosphate buffered saline (PBS, pH 7.4), lysed with lysis buffer (20 mM Tris–HCl (pH 8.0), 0.15 M NaCl, 0.1 M EDTA, 1% SDS) and stored at -70°C until use.

Proteins secreted by *A. pleuropneumoniae* into the culture media were precipitated by the addition of 0.02% deoxycholic acid (v/v) and 15% trichloroacetic acid (v/v) to the supernatant. Following overnight incubation at 4°C, the precipitate was collected by centrifugation and the pellet was washed with cold ethanol and centrifuged. After aspiration of the supernatant, the pellet was dried and dissolved in PBS.

*M. hyopneumoniae* strains were cultured to mid-log phase (medium pH range, 6.8–7.2) in ATCC medium 1699 at 37°C with continuous shaking [33]. Cell membranes were isolated by osmotic lysis and sonication. Briefly, the cell pellet was washed with PBS, resuspended in deionized water, and incubated at 37°C for 30 min. The suspensions were disrupted by sonication (thirty 2-s bursts at 200–300 W with a 2-s cooling period between each burst) and then centrifuged at 10,000 × g for 20 min at 4°C. The supernatant was centrifuged at 100,000 × g for 1 h at 4°C to collect the cell membranes [34]. The cell membrane pellet was dissolved in resuspension buffer (10 mM potassium phosphate, 50 mM NaCl, 20% glycerol (v/v)) and stored at -70°C until use.

*E. coli* DH5α and BL21 (DE3) strains (Stratagene, La Jolla, CA) were used for plasmid DNA amplification and production of recombinant proteins, respectively, and were grown in Luria-Bertani (LB) media at 37°C with continuous shaking.

### Recombinant plasmids

To prepare the pET-ApxN plasmid vector, a fragment of the *apxIIIA* gene was amplified from *A. pleuropneumoniae* genomic DNA by polymerase chain reaction (PCR) using ApxN-F and ApxN-R primers (Table 1). The PCR product was cloned into a pGEM-T Easy vector (Promega, Madison, WI). The TA-cloned vector was then digested with BamHI and XhoI and the insert was ligated into linearized pET28a(+) (Novagen, Cambridge, UK) carrying an *N*-terminal histidine tag to obtain pET-ApxN.

**Table 1:** Oligonucleotide primers used for PCR

**Primer**	**Sequence (5' to 3')**	**Accession #**	**Gene**	**Length (bp)**
ApxN-F	GG*GGATCC*GGCTACGATGTAACTAAAAATGGT	L12145	*apxIIIA*	315
	BamHI restriction sequence italicized			
ApxN-R	GG*CTCGAG*TTATTGTAAGAACTGATCCAGTTGCGG			
	XhoI restriction sequence italicized			
P97C-F	CG*GGATCC*AAACTGGATGACAACCTCCAA	U50901	*P97*	954
	BamHI restriction sequence italicized			
P97C-R	GG*CTCGAG*TTAAGGATCTCCGGATTTGCTGTCGTC			
	XhoI restriction sequence italicized			
Ap97-F	GG*CCATATG*CAAGTTAAAAAAGGCTACGATGTAAC	L12145	*apxIIIA*	333
	NdeI restriction sequence italicized			
Ap97-R	GGC*GGATCC*ATGTTTTTGTTAGAACTGATCCAGTTG			
	BamHI restriction sequence italicized			
AppCPS-F	ACTATGGCAATCAGTCGATTCAT	AY357726	*capsular polysaccharide biosynthesis*	500
AppCPS-R	CCTAATCGG AAACGCCATTCTG			
Mhyop102-F	GTCAAAGTCAAAGTCAGCAAAC	AF012905	*P102*	137
Mhyop102-R	AGCTGTTCAAATGCTTGTCC			

**Figure 1:**
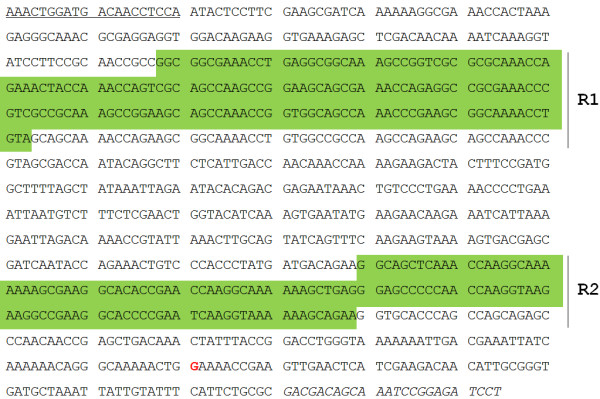
**The optimized nucleotide sequence used to express P97C in *E. coli*.** The R1 and R2 repeat regions of P97 adhesin are depicted by green backgrounds. A guanine nucleotide, shown in red font, replaces an adenine nucleotide to prevent premature termination of translation (TGA to TGG). The underlined and italicized sequences represent P97C-F and P97C-R primer binding sites, respectively.

The pET-P97C vector was constructed to express a region of the *C*-terminal portion of P97 (GenBank accession number U50901). First, a DNA fragment was synthesized, in which the nucleotide sequence of P97 was modified to produce codons preferred by *E. coli* without changing the amino acid sequence [35], and the 5’-TGA-3’ codon was replaced with 5’-TGG-3’ (Figure 1). 5’-TGA-3’ is read as tryptophan in *Mycoplasma*, but results in premature termination of translation in other eubacteria, such as *E. coli*[36]. The fragment was used to amplify a 954 bp-long product encompassing the R1 and R2 regions of P97 by PCR with P97C-F and P97C-R primers (Table 1) [37]. The product was cloned into a pGEM-T Easy vector. The TA-cloned vector was restricted with BamHI and XhoI, and the insert was ligated into linearized pET28a(+) to form pET-P97C.

The pET-Ap97 vector was used to express chimeric protein Ap97, which comprises ApxN and P97C at the *N*- and *C*-terminus, respectively. A fragment of the *apxIIIA* gene was amplified from *A. pleuropneumoniae* genomic DNA by PCR using primers Ap97-F and Ap97-R (Table 1) and cloned into a pGEM-T Easy vector. The Gly-Ser peptide linker within the chimeric protein was encoded by the sequence 5’-GGATCC-3’ within the Ap97-R primer, or by a BamHI site. The TA-cloned vector was digested with NdeI and BamHI and the insert was ligated into linearized pET-P97C to yield pET-Ap97. The 5’-TAG-3’ nucleotide sequence for a Leu residue was replaced with 5’-CAG-3’ in both Ap97-R and ApxN-R primers (further discussed in the Discussion section).

### Expression and purification of recombinant proteins

Each recombinant vector was transformed into *E. coli* BL21 (DE3) using a heat shock protocol. Selected colonies were cultured at 37°C in LB media supplemented with 100 μg/ml kanamycin. Overnight cultures (diluted 1:100) were grown until mid-log phase as indicated by an A_600_ between 0.5 and 0.7, at which point protein expression was induced for 4 h by the addition of isopropyl-β-D-thiogalactopyranoside to a final concentration of 1 mM.

Both the P97C and Ap97 proteins were purified from *E. coli* inclusion bodies. The cells were washed twice with ice-cold PBS (pH 7.4) and then lysed for 30 min in Buffer A (100 mM sodium phosphate, 10 mM Tris–HCl, 100 μg/ml lysozyme and 5 mM phenylmethanesulfonylfluoride (PMSF), pH 8.0). The lysate was centrifuged at 10,000 × g for 20 min, and the resulting pellet was resuspended by incubation for 1 h at room temperature in Buffer B (8 M urea, 10 mM Tris–HCl, 100 mM sodium phosphate and 5 mM PMSF; pH 8.0). The solubilized extracts were then loaded onto a Ni-nitrilotriacetic acid (NTA) column (Qiagen, Chatsworth, CA). The column was washed with Buffer C (8 M urea, 10 mM Tris–HCl, 100 mM sodium phosphate and 5 mM PMSF; pH 6.3) and eluted with Buffers D and E (8 M urea, 10 mM Tris–HCl, 100 mM sodium phosphate and 5 mM PMSF; pH 5.9 and 4.5, respectively) as per the manufacturer’s protocol.

To purify ApxN, the cells were washed with ice-cold PBS, lysed in Buffer F (50 mM sodium phosphate, 300 mM sodium chloride, 10 mM imidazole, 1 mg/ml lysozyme and 5 mM PMSF; pH 8.0) on ice for 30 min and sonicated on ice using six 10-s bursts at 200–300 W with a 10-s cooling period between each burst. The cell debris was pelleted by centrifugation at 10,000 × g for 20 min, and the resulting supernatant was applied to a Ni-NTA column. The column was washed twice with Buffer G (50 mM sodium phosphate, 300 mM sodium chloride, 20 mM imidazole, and 5 mM PMSF; pH 8.0). Proteins bound to the Ni-NTA column were eluted with Buffer H (50 mM sodium phosphate, 300 mM sodium chloride, 250 mM imidazole, and 5 mM PMSF; pH 8.0) as per the manufacturer’s protocol.

Purified proteins were dialyzed for 36 h at 4°C with urea or an imidazole solution diluted serially with PBS containing 150 mM sodium chloride and 5% glycerol. Protein concentrations were determined using a bicinchoninic protein assay kit (Pierce, Rockford, IL).

### Mice

All procedures for handling the mice used in this study were reviewed and approved by the Institute of Laboratory Animal Resources of Seoul National University and performed in an ethical and humane manner under veterinary supervision. Female Balb/c mice aged 6 to 8 weeks (SLC Japan, Shizuoka, Japan) were divided into groups of five animals and housed in laboratory animal facilities at the College of Veterinary Medicine, Seoul National University. The mice were kept in cages with access to water and food *ad libitum* for 2 weeks before the first immunization. The mice were anaesthetized with a mixture of 25 mg/kg Zoletil (Virbac, Carros, France) and 10 mg/kg Rompun (Bayer, Seoul, South Korea) and then immunized by subcutaneous administration of 25 μg of immunogen (Ap97, ApxN, or P97C) on Days 0, 14, 28, and 42. PBS-immunized mice were included as negative controls. Immunogens or PBS were administered in complete Freund’s adjuvant on Day 0 and in incomplete Freund’s adjuvant on Days 14, 28, and 42. Freund’s adjuvants are potent oil-based adjuvants that are commonly used in animal research [38].

Serum samples were obtained by retro-orbital bleeding under anesthesia before immunization on Days 0, 14, 28, and 42. The mice were sacrificed by cardiac puncture 3 weeks after the final immunization (Day 63), and splenectomies were performed to enable the primary culture of splenocytes.

### Splenocytes and cytokine analysis

The spleens of mice immunized with Ap97, ApxN, and P97C vaccines were aseptically removed, and single cell suspensions were prepared by passing the organ through a 200-μm nylon mesh sieve. Splenocytes were resuspended in ACK buffer (0.15 M ammonium chloride, 10 mM potassium bicarbonate, and 0.1 mM EDTA, pH 7.4), washed twice with RPMI 1640 media, and cultured in RPMI 1640 media supplemented with 10% fetal bovine serum, 1 mM sodium pyruvate, 2 mM L-glutamine, 100 U/ml penicillin, 100 μg/ml streptomycin and 50 μM β-mercaptoethanol at 37°C under 5% CO_2_ in a humidified atmosphere. For analysis of cytokine production, splenocytes (4 × 10^6^ cells/ml) were re-stimulated for 72 h with 1.25 μg/ml of Ap97, ApxN, or P97C dissolved in PBS. At the end of re-stimulation, cell culture supernatants were harvested and analyzed for IFN-γ and IL-4 using a bead-based multiplex assay kit (Procarta cytokine assay, Panomics, Fremont, CA), as per the manufacturer’s protocol.

### Enzyme-linked immunosorbent assay *(ELISA)*

The immuno-reactivity of the mouse serum samples against ApxN and P97C was measured using an indirect ELISA. In brief, the wells of a 96-well plate were coated with 0.5 μg of ApxN or P97C in 50 μl of carbonate buffer (pH 9.6) overnight at 4°C. The wells were washed three times with PBS containing 0.05% Tween 20 (PBS-T), blocked with 5% skim milk for 2 h at 37°C, washed with PBS-T, and then incubated with serially diluted serum samples for 2 h at 37°C. The wells were washed and incubated with peroxidase-conjugated anti-mouse IgG, IgG1, or IgG2a (diluted 1:1,000 in PBS-T) for 1 h at 37°C. After washing, the color was developed with *o*-phenylenediamine (Amresco, Solon, OH) and 3 M HCl was used to stop the reaction. The absorbance was measured at 450 nm in a micro-plate spectrophotometer (Molecular Devices, Sunnyvale, CA). The cut-off values for the assay were calculated as the mean specific optical density (OD) plus three standard deviations (SDs) using serum samples of non-immunized mice (Day 0) assayed at a 1:100 dilution. Titers were established as the reciprocal of the last serum dilution yielding an OD higher than the cut-off.

### Gel electrophoresis and western blot analysis

Protein samples were separated by sodium dodecyl sulfate-polyacrylamide gel electrophoresis (SDS-PAGE; 8–10% acrylamide) using a minigel apparatus (Bio-Rad, Hercules, CA) and stained with Coomassie blue. For western blot analysis, the proteins in the SDS-PAGE gels were transferred to a Protran nitrocellulose membrane (Whatman, Dassel, Germany) in a blotting apparatus (Bio-Rad). The blots were blocked by incubation with 5% skim milk for 1 h and then incubated with antisera from mice or pigs (diluted 1:500) overnight at 4°C. The blots were then incubated with peroxidase-conjugated anti-mouse IgG or anti-pig IgG (MP Biomedicals, Solon, OH) secondary antibodies (diluted 1:5,000) for 1 h and developed with chemiluminescence reagents (Ab Frontier, Seoul, Korea).

### Pigs

Ten 3-week-old male pigs weighing 4–6 kg were obtained from a pathogen-free farm. Pigs were randomly divided into two groups, housed in pens with slatted floors and rubber mats in separate rooms, and provided with water and food *ad libitum*. Pigs were shown to be free of pathogens by PCR analysis of nasal swabs. A 500-bp fragment of the gene encoding a capsular polysaccharide from *A. pleuropneumoniae* serotype 2 was amplified using the AppCPS-F and AppCPS-R primers (Table 1), and a 137-bp fragment of the P102 gene from *M. hyopneumoniae* was amplified using the Mhyop102-F and Mhyop102-R primers.

After a week of acclimatization, both groups of pigs was immunized intramuscularly with either 700 μg of Ap97 adsorbed onto aluminum hydroxide, a standard adjuvant in clinical veterinary vaccines [39], or 2 ml PBS containing aluminum hydroxide as the negative control (Day 0). A booster was inoculated on Day 14 in a similar manner. Blood samples for immuno-serological analysis were drawn by puncture of the vena cava cranialis on Day 24 after the first immunization, and serum was obtained by centrifugation for 15 min at 1,500 × g. In challenge treatments performed following blood collection, pigs were inoculated with 7 ml culture media containing 2 × 10^8^ colony-forming units of *A. pleuropneumoniae* and 1.5 × 10^8^ color-changing units of *M. hyopneumoniae* by intranasal and intratracheal administration, respectively. Clinical signs such as rectal temperature, coughing, dyspenia, tachypnea, depression, and nasal discharge were monitored and recorded 2 weeks after challenge (Day 38). Pigs were euthanized using electric current and complete necroscopies were performed 21 days after challenge (Day 45). The macroscopic lung lesion score was determined as described previously [15,40].

### Statistics

Statistical analyses were performed using SPSS 15.0 (SPSS, Chicago, IL). Data were expressed as the mean ± SD. The unpaired Student’s *t*-test was used to compare antibody titers and macroscopic lung lesion scores. The paired Student’s *t*-test was used to evaluate differences in cytokine production. Data were considered significantly different when *P* was < 0.05.

## Results

### Production of recombinant proteins

**Figure 2:**
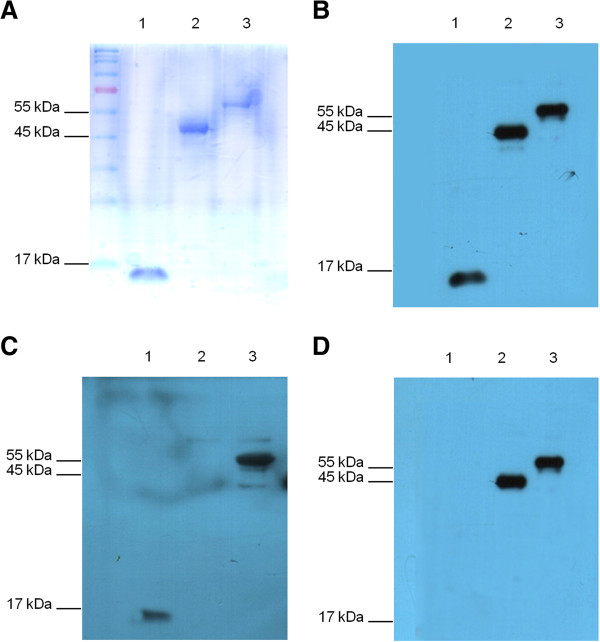
**Expression and purification of ApxN, P97C, and Ap97 recombinant proteins. A**. Coomassie blue-stained SDS-PAGE gel showing ApxN, P97C, and Ap97 purified from *E. coli* BL21 using His-tag affinity chromatography. The molecular masses of ApxN (lane 1), P97C (lane 2), and Ap97 (lane 3) were approximately 15, 45, and 60 kDa, respectively, which were equivalent to the expected values. **B**, **C**, and **D**. Western blot analysis of ApxN, P97C, and Ap97 using antisera from mice immunized with Ap97 **(B)**, ApxN **(C)**, or P97C **(D)** vaccines. The mouse antisera were obtained 3 weeks after the last immunization (Day 63).

SDS-PAGE analysis of proteins purified after expression in *E. coli* using pET-ApxN, pET-P97C, and pET-Ap97 vectors revealed prominent bands at approximately 15 kDa, 45 kDa, and 60 kDa, respectively (Figure 2A). These values corresponded to the expected molecular weights of ApxN, P97C, and Ap97 (a chimeric protein comprising ApxN and P97C). These findings demonstrate that the recombinant proteins were expressed as expected.

### Immunogenicity of recombinant proteins in mice

Antiserum generated in mice immunized with the Ap97 vaccine was used to test the ability of the chimeric protein to induce the production of antibodies specific for each of its component subunits, ApxN and P97C, as well as to the whole chimeric protein (Figure 2B). Antiserum against the Ap97 vaccine reacted well with ApxN, P97C, and Ap97, demonstrating that immunization with Ap97 induces the production of antibodies that bind to all three recombinant proteins. Thus, Ap97 retained the antigenic characteristics of its two individual components. Antisera from mice immunized with the ApxN and P97C vaccines reacted with Ap97, and with ApxN and P97C, respectively; however, antisera from mice immunized with ApxN did not cross react with P97C and vice versa (Figure 2C and D).

**Figure 3:**
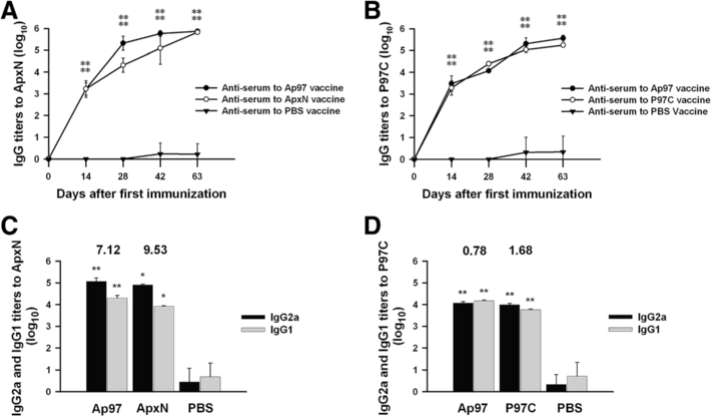
**Serum titers of total IgG, IgG1, and IgG2a isotype antibodies against ApxN and P97C in mice immunized with ApxN, P97C, and Ap97 vaccines.** Blood samples were obtained at 0, 14, 28, 42, and 63 Days after the first immunization. **A**. Total IgG titers against ApxN. **B**. Total IgG titers against P97C. **C**. IgG isotype titers against ApxN. **D**. IgG isotype titers against P97C. IgG1 and IgG2a titers were evaluated at 3 weeks after the last immunization (Day 63). The numbers above the columns indicate the mean IgG2a/IgG1 ratios **(C and D)**. Data represent the mean ± SD (n = 5). *P < 0.05 and **P < 0.01, significantly different from the corresponding anti-PBS serum controls (unpaired *t*-test).

Immunization with the Ap97 and ApxN vaccines induced vigorous IgG responses to ApxN in mice. The IgG log titers reached 3.22 and 3.22 on Day 14 after the first immunization, and 5.87 and 5.82 at 3 weeks after the last immunization (Day 63) with Ap97 and ApxN, respectively, which were significantly higher than those recorded for antisera obtained from mice given PBS (Figure 3A; *P* < 0.01). Immunization with the Ap97 and P97C vaccines also increased the levels of anti-P97C IgG antibodies in the mice, with log titers reaching 5.55 and 5.23 at 3 weeks after the last immunization (Day 63), respectively; these values are also higher than those for the antisera obtained from mice given PBS (Figure 3B; *P* < 0.01).

The levels of both the IgG1 and IgG2a isotypes against ApxN and P97C were measured in sera collected 3 weeks after the last immunization (Day 63; Figure 3C and 3D). The levels of both isotypes against ApxN and P97C were higher than those in the corresponding PBS-treated controls (*P* < 0.01 or *P* < 0.05), indicating mixed Th1-Th2 responses. However, the mean ratios of IgG2a/IgG1 against ApxN were 7.12 and 9.53 in antisera from mice given the Ap97 and ApxN vaccines, respectively, indicating the predominance of IgG2a production over IgG1 and, thus, a Th1-biased response to ApxN. By contrast, the IgG2a/IgG1 ratios to P97C were 0.78 and 1.68 in the antisera from mice given the Ap97 and P97C vaccines, respectively. This demonstrates that Ap97 and P97C induced relatively balanced IgG2a and IgG1 responses to P97C.

### Cytokine secretion induced by recombinant proteins

**Figure 4:**
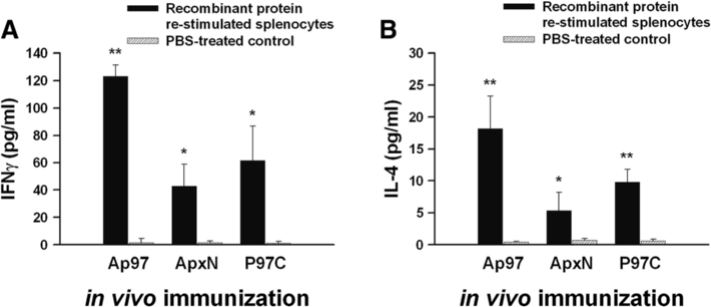
**Cytokine profiles of splenocytes obtained from immunized mice.** Mice were primed with Ap97, ApxN, or P97C vaccines. Splenocytes were isolated from each mouse and then re-stimulated with Ap97, ApxN, or P97C dissolved in PBS. At the end of re-stimulation, cell culture supernatants were analyzed for IFN-γ **(A)** and IL-4 **(B)**. Data represent the mean ± SD (n = 4). *P < 0.05 and **P < 0.01, significantly different from the corresponding PBS-treated controls (paired t-test).

Re-stimulated splenic mononuclear cells (splenocytes) from mice immunized with Ap97, ApxN, and P97C vaccines were assayed for the production of the IFN-γ and IL-4 (Figure 4). The concentrations of IFN-γ, a Th1 cytokine, in the culture medium of splenocytes re-stimulated *in vitro* with Ap97, ApxN, and P97C were 123.02 ± 8.23 pg/ml, 42.89 ± 15.81 pg/ml, and 61.51 ± 25.36 pg/ml, respectively. Those levels were 80.9, 29.4, and 50.8 times higher, respectively, than those re-stimulated with PBS (control) (Figure 4A; *P* < 0.01 or *P* < 0.05). The concentrations of IL-4, a Th2 cytokine, produced by splenocytes re-stimulated *in vitro* with Ap97, ApxN, and P97C were 18.21 ± 5.08 pg/ml, 5.36 ± 2.83 pg/ml, and 9.77 ± 2.04 pg/ml, respectively. Those levels were 43.4, 7.66 and 16.3 times higher, respectively, than those in the PBS controls (Figure 4B; *P* < 0.01 or *P* < 0.05). Stimulation with concanavalin A, a potent T cell stimulator, also induced splenocytes (including the controls) to produce these two cytokines (data not shown). These findings indicate that all three recombinant proteins triggered the production of both IFN-γ and IL-4 by splenocytes, suggesting the induction of mixed Th1-Th2 immune responses.

### Reaction of ApxIII and P97 with mouse antisera to the recombinant proteins

**Figure 5:**
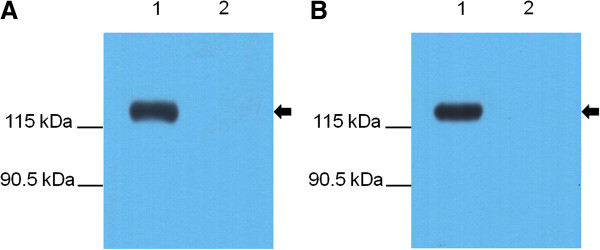
**Recognition of *A. pleuropneumoniae *ApxIII by antisera from mice immunized with the Ap97 or ApxN recombinant proteins.** Western blot analyses were performed with ApxN-vaccinated **(A)** and Ap97-vaccinated **(B)** mouse sera for proteins precipitated from *A. pleuropneumoniae* 1536 culture media (Lane 1) and control media (Lane 2). Arrows indicate the target band size.

Western blots were performed to examine the reaction of native ApxIII with mouse antisera raised against the Ap97 and ApxN vaccines (Figure 5). A protein sample precipitated from the culture medium of *A. pleuropneumoniae* was used for the analysis (see Methods). Antisera generated in mice immunized with the ApxN and Ap97 vaccines reacted with a precipitated protein that was approximately 120 kDa in size, which corresponds to the molecular weight of ApxIII [41], whereas no protein signal was detected in the control media, indicating that the antibodies generated in response to vaccination with Ap97 and ApxN readily bind to the ApxIII excreted by *A. pleuropneumoniae*. These data suggest that Ap97, as well as ApxN, possess the antigenic characteristics of the ApxIII protein, which are recognized by the mouse immune system.

**Figure 6:**
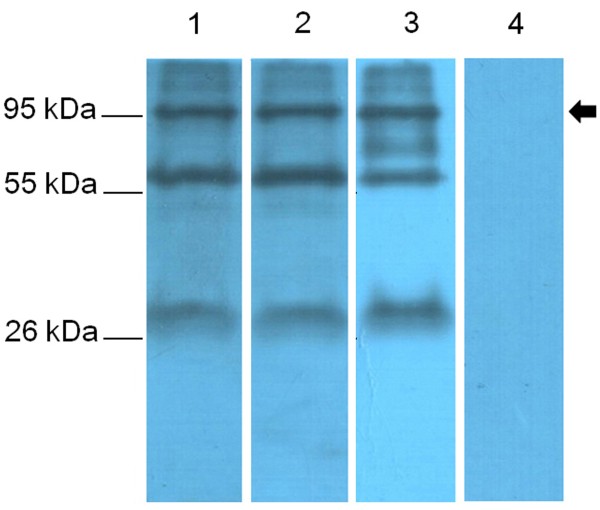
**Recognition of *M. hyopneumoniae *P97.** The cell membrane fraction from *M. hyopneumoniae* was subjected to western blot analysis with Ap97-vaccinated mouse serum (Lane 1), P97C-vaccinated mouse serum (Lane 2), the serum of a pig experimentally infected with *M. hyopneumoniae* (Lane 3), and PBS-vaccinated control mouse serum (Lane 4). An arrow indicates the target band size.

Antisera from mice immunized with the Ap97 and P97C vaccines were also assayed for reaction with P97, found in the membrane fraction of *M. hyopneumoniae* (Figure 6). The antisera detected bands of approximately 97 KDa, the predicted size of P97. These data suggest that both Ap97 and P97C retain the antigenic characteristics of native P97. In addition, serum from a pig artificially infected with *M. hyopneumoniae* reacted with the 97 kDa protein. All three antisera also reacted with two other proteins (30 and 60 kDa), and the pig antiserum detected an additional band at 70 kDa. These protein bands, including P97, were not recognized by the control serum.

### Immunogenicity of Ap97 in pigs

**Figure 7:**
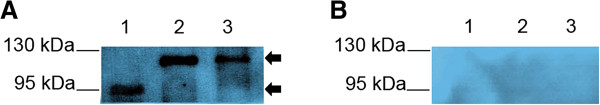
**Recognition of *M. hyopneumoniae *P97 and *A. pleuropneumoniae *ApxIII by serum isolated from pigs immunized with the Ap97 vaccine. A**. Western blot analysis of the cell membrane fraction of *M. hyopneumoniae* (Lane 1) as well as TCA-precipitated proteins from the culture media (Lane 2) and cell lysate (Lane 3) of *A. pleuropneumoniae* was performed with Ap97-vaccinated pig serum. **B**. Western blot analysis of the protein samples described in panel **A** was performed with PBS-vaccinated pig serum. Arrows indicate target band sizes.

We also assessed the reactivity of Ap97 vaccine-treated pig serum against native P97 and ApxIII (Figure 7). Antiserum to the Ap97 vaccine recognized protein bands of the expected molecular masses; 97 kDa in the cell membrane fraction of *M. hyopneumoniae* and 120 kDa in the culture media and cell lysate of *A. pleuropneumoniae*. The data are representative of six experiments, in which independent antiserum samples were used. In addition, the serum from pigs treated with PBS did not react with P97 or ApxIII. These data suggest that antibodies induced in pigs vaccinated with Ap97 efficiently react with P97 and ApxIII, and that both domains of the Ap97 protein are immunogenic in pigs.

### Protective efficacy of Ap97 in pigs

**Figure 8:**
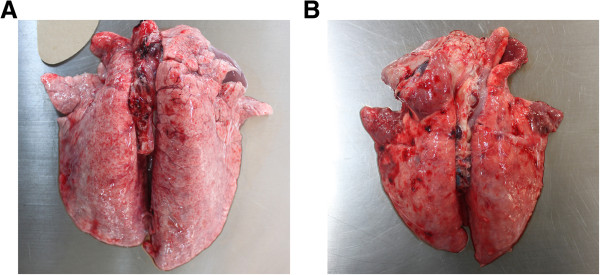
**Ventral surfaces of lungs from Ap97 (A)- and PBS (B)-vaccinated pigs at day 21 post-challenge with *A. pleuropneumoniae *** **and *M. hyopneumoniae*.**

Since pigs immunized with Ap97 produced antibodies against P97 and ApxIII (Figure 7), the pigs were directly subjected to challenge with *M. hyopneumoniae* and *A. pleuropneumoniae*. The PBS vaccine-treated control pigs that were challenged with *A. pleuropneumoniae* and *M. hyopneumoniae* showed an increased body temperature and developed clinical symptoms, including coughing, dyspnea, depression, and nasal discharge (Table 2). Postmortem examination revealed that all the PBS-immunized pigs had numerous gross pathological changes linked to *A. pleuropneumoniae* and *M. hyopneumoniae* (Figure 8). The lungs of the PBS-vaccinated pigs developed well-demarcated dark red areas of cranioventral consolidation, which are lesions consistent with mycoplasmal pneumonia, and also developed multifocal hemorrhages and fibrinous pleurisy that are classic presentation of *A. pleuropneumoniae* disease [1,3,14,15]. In contrast, no remarkable clinical signs or lung lesions were observed in the Ap97-vaccinated group (with the exception of mild coughing in one pig).

**Table 2:** **Protection of pigs with Ap97 vaccine against *A. pleuropneumoniae *** **and *M. hyopneumoniae *** **infection**

**Immunogen**	**Challenge**	**Body temperature**^**a**^	**# of pigs with clinical signs / # of pigs challenged**				**Lung lesion score**^**b **^**(Mean ± SD)**
			**Coughing**	**Dyspnea (tachypnea)**	**Depression**	**Nasal discharge**	
Ap97	*A. pleuropneumoniae* and *M. hyopneumoniae*	-,-,-,-,-,-	1/6	0/6	0/6	0/6	2.00 ± 0.71^*^
PBS	*A. pleuropneumoniae* and *M. hyopneumoniae*	+,+,++,++,++,++	6/6	5/6	5/6	6/6	62.30 ± 4.38

^a^Maximum measured rectal temperature. –, ≤ 39.5°C; +, 39.5 to 42°C; ++, ≥ 42°C.

^b^Total area (0–100%) of the lung affected by any type of pneumonia lesion [51]. Data are presented as the mean ± SD (n = 6). *P < 0.01, significantly different from the corresponding PBS-treated group (unpaired *t*-test).

## Discussion

There is a high demand for multi-disease and multivalent vaccines in the animal health industry. However, combining vaccine antigens causes a variety of problems, including interference between the various components, antigenic competition, and carrier-specific epitope suppression [42-44]. Thus, combined formulations must be shown to be both safe and effective before licensure. Numerous examples of immunological interference have been observed in laboratory tests and clinical trials [45]. One relevant example involves a study of immunity against *A. pleuropneumoniae* in swine, in which the protective efficacy of an ApxI/ApxII vaccine was lost when it was supplemented with PalA, an outer membrane protein derived from the pathogen [11].

The first 160 residues of the ApxIII *N*-terminus are involved in its cytotoxicity and pro-apoptotic activity, and convalescent serum collected from a pig infected with *A. pleuropneumoniae* reacted with the *N*-terminus [46]. Based upon these findings, the region encompassing residues 21–131 of ApxIII was used to construct Ap97. In preliminary studies, the first 20 residues of ApxIII were also included, but the expression efficiency was very low (data not shown). This could be due to the inclusion of two under-represented Arg codons (AGG and AGA) at residues 14 and 20, respectively. In *E. coli*, AUA (Ile), CUA (Leu), and AGA, AGG, or CGA (Arg), represent less than 8% of their corresponding population of codons [47]. Another rarely-observed codon, CUA, at residue 124 of ApxIII was replaced with CUG using an Ap97-R primer that contains a single base substitution (Table 1). This was also the case for the expression of ApxN.

The expression of recombinant proteins, especially chimeric proteins, can result in aberrant tertiary folding of the expressed protein; correct folding is critical for maintaining the antigenic characteristics. It was not established whether or not both components of Ap97 protein possessed the correct tertiary structure. However, Ap97 retained the antigenic properties of its two component proteins, ApxN and P97C (Figure 2), and antisera from mice and pigs immunized with Ap97 readily reacted with native ApxIII and P97 from *A. pleuropneumoniae* and *M. hyopneumoniae*, respectively (Figures 5, 6, 7). In addition, immunization with the Ap97 vaccine induced humoral and cellular immune responses in mice (Figures 3 and 4), and pigs immunized with the Ap97 vaccine were protected against challenge by *A. pleuropneumoniae* and *M. hyopneumoniae* (Table 2). These findings suggest that Ap97 contains antigenic epitopes similar to those of native ApxIII and P97.

IL-4 activates B cells and induces class switching to the IgG1 isotype (a Th2 type immune response), while IFN-γ induces IgG2a responses (Th1 type) [48,49]. A Th1 antibody response is involved in protection from infection, while a Th2 response is helpful for clearing the infection. Thus, an ideal vaccine should enhance Th1 responses while concomitantly maintaining Th2 responses. Ap97 induced secretion of both Th1 and Th2 cytokines by splenocytes (Figure 4). Along with an increase in cytokine levels, the levels of both the IgG1 and IgG2a isotypes specific for ApxN and P97C were also increased in the sera of Ap97-immunized mice (Figure 3). These indicate that Ap97 induced a mixed Th1-Th2 response. P97C induced balanced IgG1 and IgG2 responses in mice (Figure 3). By contrast, ApxN induced IgG2a more than IgG1, indicating a Th1-biased response to ApxN. This difference in the responses elicited by the two components of Ap97 is interesting; however, the underlying mechanism is unclear. Previous studies indicate that the pattern of IgG isotypes induced by protein vaccines depends on the adjuvants and protein antigens used [50-52].

Of the three recombinant proteins, Ap97 induced the greatest concentrations of IFN-γ and IL-4 in the culture media of re-stimulated splenocytes (Figure 4). Notably, the concentration of IFN-γ in the media from Ap97-stimulated cells was a bit higher than the sum of the cytokine concentrations in the media from ApxN and P97C-stimulated cells. This was also the case for IL-4. These suggest that both components of Ap97 do not interfere each other on cytokine induction.

The membrane fraction of *M. hyopneumoniae* contained not only P97 but different-sized proteins, which were recognized by serum from a pig artificially infected with *M. hyopneumoniae* as well as antisera from mice immunized with the Ap97 and P97C vaccines (Figure 6). Based on previous studies that identified different-sized cleavage products of P97, the non-97 kDa proteins may represent cleavage products of P97 [22,27,53].

## Conclusions

The chimeric protein, Ap97, was both immunogenic and effective as a vaccine under the conditions of these experiments. To the best of our knowledge, this is the first study to demonstrate the prevention of infection by *A. pleuropneumoniae* and *M. hyopneumoniae* using a multi-disease subunit vaccine. The only previous report related to multi-disease vaccination against these two pathogens was based on immunization with a live attenuated strain of *A. pleuropneumoniae*, which was engineered to express *M. hyopneumoniae* P36 [54]. While the results of this study are limited to two proteins derived from these pathogens, it will be interesting to evaluate the potential of other proteins derived from these microorganisms, especially ApxI, ApxII, and ApxIV toxins from various *A. pleuropneumoniae* serotypes, to contribute to the development of safer and more effective multi-disease vaccines.

## Competing interests

The authors declare that they have no competing interests.

## Authors’ contributions

SHL and SL performed research; SHL, CC, and DYR designed research and analyzed data; and SHL and DYR wrote this manuscript. All authors read and approved the final manuscript.
